# Induction of osteogenic markers in differentially treated cultures of embryonic stem cells

**DOI:** 10.1186/1746-160X-4-10

**Published:** 2008-06-10

**Authors:** Jörg Handschel, Karin Berr, Rita A Depprich, Norbert R Kübler, Christian Naujoks, Hans-Peter Wiesmann, Michelle A Ommerborn, Ulrich Meyer

**Affiliations:** 1 Department for Cranio- and Maxillofacial Surgery, Heinrich-Heine-University Düsseldorf, Moorenstr. 5, 40225 Düsseldorf, Germany; 2 Department for Cranio- and Maxillofacial Surgery, Westfälische-Wilhelms-Universität Münster, Waldeyerstr. 30, 48149 Münster, Germany; 3 Department for Operative and Preventive Dentistry and Endodontics, Heinrich-Heine-University Düsseldorf, Moorenstr. 5, 40225 Düsseldorf, Germany

## Abstract

**Background:**

Facial trauma or tumor surgery in the head and face area often lead to massive destruction of the facial skeleton. Cell-based bone reconstruction therapies promise to offer new therapeutic opportunities for the repair of bone damaged by disease or injury. Currently, embryonic stem cells (ESCs) are discussed to be a potential cell source for bone tissue engineering. The purpose of this study was to investigate various supplements in culture media with respect to the induction of osteogenic differentiation.

**Methods:**

Murine ESCs were cultured in the presence of LIF (leukemia inhibitory factor), DAG (dexamethasone, ascorbic acid and β-glycerophosphate) or bone morphogenetic protein-2 (BMP-2). Microscopical analyses were performed using von Kossa staining, and expression of osteogenic marker genes was determined by real time PCR.

**Results:**

ESCs cultured with DAG showed by far the largest deposition of calcium phosphate-containing minerals. Starting at day 9 of culture, a strong increase in collagen I mRNA expression was detected in the DAG-treated cells. In BMP-2-treated ESCs the collagen I mRNA induction was less increased. Expression of osteocalcin, a highly specific marker for osteogentic differentiation, showed a double-peaked curve in DAG-treated cells. ESCs cultured in the presence of DAG showed a strong increase in osteocalcin mRNA at day 9 followed by a second peak starting at day 17.

**Conclusion:**

Supplementation of ESC cell cultures with DAG is effective in inducing osteogenic differentiation and appears to be more potent than stimulation with BMP-2 alone. Thus, DAG treatment can be recommended for generating ESC populations with osteogenic differentiation that are intended for use in bone tissue engineering.

## Background

Facial trauma or tumor surgery in the head and face area often lead to massive destruction of the facial skeleton [1]. The reconstruction of damaged or lost bone is a clinical challenge in modern reconstructive surgery. The repair of bone defects still poses a significant problem for many clinicians. In the early decades of bone reconstruction surgeons used artificial tissue substitutes containing metals, ceramics, and polymers to maintain skeletal function [2]. These artificial materials have facilitated surgeons to restore the form and – to some extent – the function of defective bones. Nevertheless, these artificial materials have specific disadvantages, and thus encouraged surgeons to develop alternative approaches including cell-based devices. Transplantation of autografts is a frequently used treatment strategy in routine clinical practice and has gained the "gold standard" in bone reconstructive surgery, despite donor site morbidity and donor shortage [3].

Modern cell-based bone reconstruction techniques may offer new therapeutic opportunities for the repair of bone damaged by disease or injury. Generally, the combination of scaffolds, bioactive factors, and living cells provides a surgically implantable product for use in tissue regeneration and functional restoration [4,5]. Numerous attempts were undertaken with various success to restore bone defects by various biomaterials alone [6-10] or in combination with bioactive cytokines such as bone morphogenetic protein (BMP)-7, BMP-2 or BMP-2-mutants [11,12]. Cell-based strategies in bone tissue engineering use different cell sources including autologous cells as well as allogenic and xenogenic cells [13-16]. There are some reports that use totipotential embryonic stem cells in tissue engineering of bone [17,18].

Embryonic stem cells (ESCs) are routinely derived from the inner cell mass of blastocysts and represent pluripotential embryonic precursor cells that give rise to all cell types in the developing organism. ESCs have historically been maintained in co-culture with mitotically inactive fibroblasts [19-21]. This co-culture system is unnecessary if the medium is supplemented with leukemia inhibitory factor (LIF) [22,23]. In the absence of LIF embryonic stem cells will differentiate into a morphologically mixed cell population expressing features of endoderm and mesoderm lineages [24]. By definition ESCs have the potential to differentiate into osteogenic cells under selective culture conditions. Specifically, it has been shown by various investigators that ESCs can differentiate into osteogenic cells under selective culture conditions [17,18,25]. However, it is unclear which medium is most suitable to initiate osteogenic differentiation. BMP-2 and a mixture of dexamethasone, ascorbic acid and β-glycerophosphate (DAG) are good candidates [19,25]. Thus, we examined the time-dependent expression of the osteoblastic markers osteopontin [26], collagen I [27], alkaline phosphatase [28], and osteocalcin [29] in ESC cells.

## Methods

### Culture of ESCs with biomaterials

Feeder-independent murine ESCs were derived from the inner cell mass of blastocysts extracted from C57BL/6 mice. The ESCs were kindly provided by K. Pfeffer (Institute for Microbiology, Heinrich-Heine-University, Germany). The cells were tested to be positive for the stem cell marker Pouf1 (alias Oct4) and Foxd3 [30] (data not shown). A total number of 1.5 × 10^6 ^cells per petri dish (10 cm in diameter) were cultured in Dulbecco's Eagle medium (DMEM). The medium was supplemented with 5 mM glutamine, 100 units/ml penicillin, 100 μg/ml streptomycin, 50 μM 2-mercaptoethanol and 15% fetal calf serum (FCS). The ESCs were divided into four groups and cultured for 25 days as follows: group I; control, supplemented with LIF to prevent differentiation, group II; no additional supplement, group III; supplemented with BMP-2 (10 ng/ml), and group IV; supplemented with DAG (dexamethasone (0.1 μM), ascorbic acid (50 μM) and β-glycerophosphate (10 mM).

### Microscopical analyses

To detect mineralization in the differently treated cell cultures, the cells were washed two times with PBS (phosphate-buffered saline) before fixation with 3% glutardialdehyde in PBS for 30 minutes. The cells were washed with distilled water and incubated in 5% silver nitrate (Sigma Aldrich) for 1 hour. The cells were washed again with distilled water. A solution of 5% sodium carbonate and 10% formaldehyde was added for 2 minutes before the cells were washed again and fixed with 1% sodium thiosulfate. Calcium-phosphate deposits stained black [31,32].

### Quantitative real time PCR

Quantitative real time PCR was employed to assess the influence of the biomaterials on gene expression. Total RNA was isolated from specimens using the RNeasy Mini Kit (Qiagen, Hilden, Germany) according to the manufacturer's instructions. For cDNA synthesis 800 ng total RNA was used as a template for Superscript II (Invitrogen, Paisley, UK) and OligodT-Primers (Peqlab, Erlangen, Germany) in a total volume of 20 μl. Amplification was performed with 1 μl of cDNA and the following specific primer pairs (MWG-Biotech AG, Ebersberg, Germany): CD34; 5'-CACAGAACTTCCCAGCAAACTC-3' and 5'-CATGTTGTCTTGCTGAATGGCC-3', osteopontin; 5'-CCCGGTGAAAGTGACTGATT-3' and 5'-TTCTTCAGAGGACACAGCATTC-3', osteocalcin; 5'-GCCCTGAGTCTGACAAAGGTA-3' and 5'-GGTGATGGCCAAGACTAAGG-3', collagen type I; 5'-AAGGGGTCTTCCTGGTGAAT-3' and 5'-GGGGTACCACGTTCTCCTC-3', alkaline phosphatases; 5'-AAGGCTTCTTCTTGCTGGTG-3' and 5'-GCCTTACCCTCATGATGTCC-3', and GAPDH; 5'-CAATGAATACGGCTACAGCAAC-3' and 5'-AGGGAGATGCTCAGTGTTGG-3'. For quantitative real time PCR the iCycler Thermal Cycler Base (Bio-Rad Laboratories GmbH, München, Germany) and qPCR MasterMix, No Rox, #RT-QP2X-03NR (Eurogentec, Köln, Germany) was used. The increase in reaction products during PCR was monitored by measuring the increase in fluorescence intensity caused by the binding of SYBR green to double-stranded DNA that accumulated during PCR cycles. Reaction mixtures were set up as suggested by the manufacturer. Threshold cycle values of target genes were standardized against GAPDH expression and normalized to the expression in the control culture (group I). All real time experiments in this study have been performed with regard to the publication of Pfaffl [33]. We have applied the mathematical model given there to eliminate deviations due to sample preparation. In order to apply this model it is necessary to choose a reference gene (e.g. GAPDH) for calculating relative expression levels. The quantitative real time PCR was performed in samples obtained at day 5, 9, 11, 13, 15, 17, 19, 21, 23, and 25 of culture, respectively. Following PCR agarose-gel electrophoresis was performed using β-actin as a reference.

## Results

**Figure 1:**
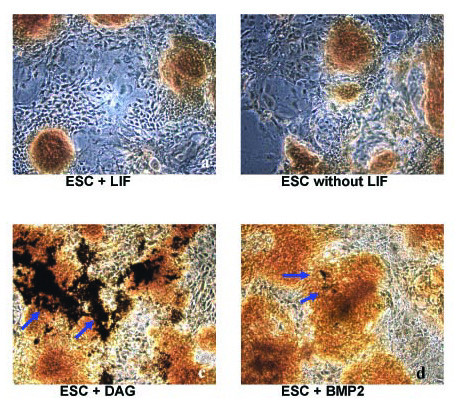
**Mi**neral deposition at day 14 in differently treated embryonic stem cells (ESCs)**.** Cells were exposed to (a) LIF (leukemia inhibitory factor), (b) without LIF, (c) DAG (dexamethasone, ascorbic acid and β-glycerophosphate) or (d) BMP-2. Shown are von Kossa stainings with arrows pointing to the deposition of calcium phosphate-containing minerals that stained in black.

In ESC cultures supplemented with DAG we found the largest deposition of calcium phosphate-containing minerals, as judged by von Kossa staining (Fig. 1). ESCs cultured in the presence of BMP-2 exhibited less mineralization, and there were no signs of mineral deposition in unstimulated control cells or cells stimulated with LIF.

**Figure 2:**
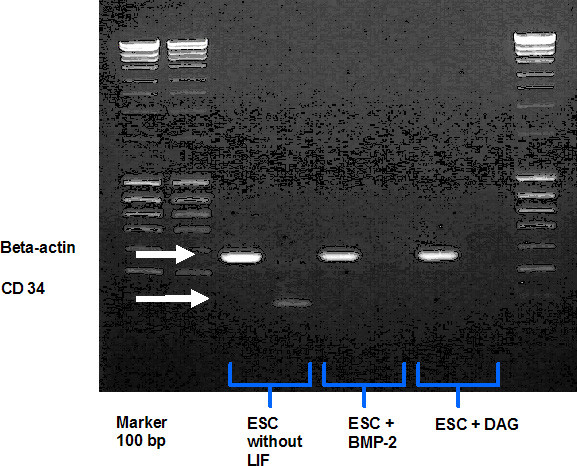
**Results from qualitative PCR showing amplification of the hematopoetic stem cell marker CD34 in ESC cells treated (a) without LIF, (b) with BMP-2, or (c) DAG**. Beta-actin was used as control.

In order to assess the differentiation of ESCs cultured under different conditions, we used the hematopoetic stem cell marker CD34. Only in ESC cultures without any additional stimulus (ESCs without LIF) the expected amplicon appeared in agarose-gel electrophoresis. ESCs which were differentiated with BMP-2 or DAG have downregulated this marker (Fig. 2).

**Figure 3:**
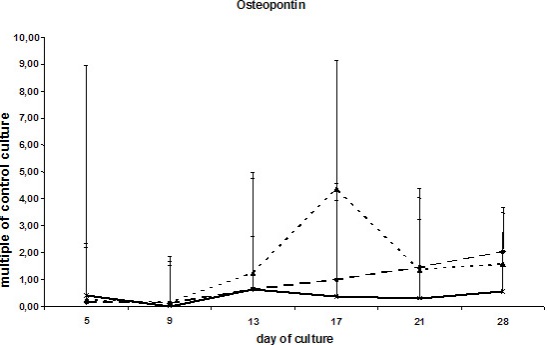
mRNA levels for osteopontin in ESCs cultured with DAG , BMP-2  or without additional supplements (ESC without LIF) . Values are calculated as multiples of the transcription level of the control culture (ESC with LIF). Shown as mean values and standard deviations normalized to the expression of GAPDH.

Next the kinetics of gene expression in ESCs during differentiation and matrix formation were evaluated. The values were plotted as a multiple of the expression in the control group (ESCs with LIF). Expression of osteopontin was reduced in ESC treated with LIF as compared to all other samples (without LIF, with BMP-2 or with DAG). The low level of osteopontin mRNA synthesis persisted in the presence of DAG, and in BMP-2-treated cells showed a steep increase after 2.5 weeks of culture. ESCs without LIF showed similar expression rates as the DAG group (Fig. 3).

**Figure 4:**
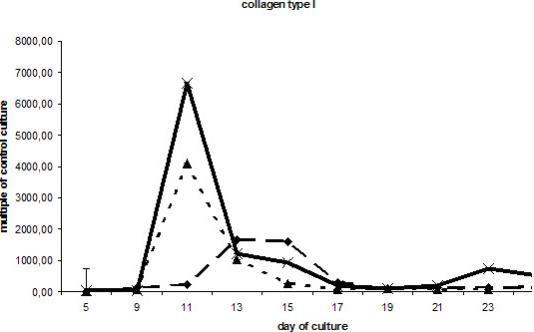
Expression of collagen I transcripts in differentially treated ESCs cultures: DAG , BMP-2  and without additional supplements (ESC without LIF) . Values are calculated as multiples of the transcription level of the control culture (ESC with LIF) and shown as mean values and standard deviations after normalisation against GAPDH.

Starting at day 9 of culture, a strong increase in collagen I expression was recorded in the DAG culture, which was paralleled to a lesser extent by the collagen expression in the BMP-2-treated cells. After three weeks of culture the expression level of collagen I mRNA was similar in all groups of the differentially treated cells (Fig. 4). Only the DAG culture showed a second but smaller increase at day 23.

**Figure 5:**
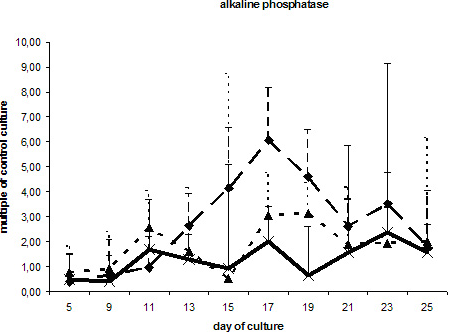
mRNA levels of alkaline phosphatase in ESCs cultured with DAG , BMP-2  and without additional supplements (ESC without LIF) . Data are presented as in Fig. 3.

**Figure 6:**
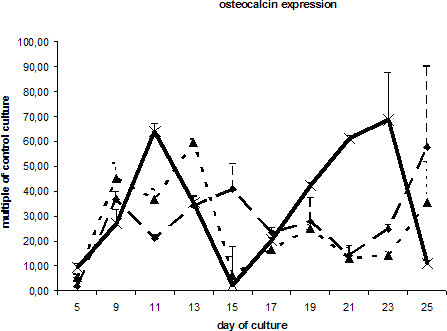
mRNA levels of osteocalcin in ESCs cultured with DAG , BMP-2  and without additional supplements (ESC without LIF) . Data are presented as in Fig. 3.

**Figure 7:**
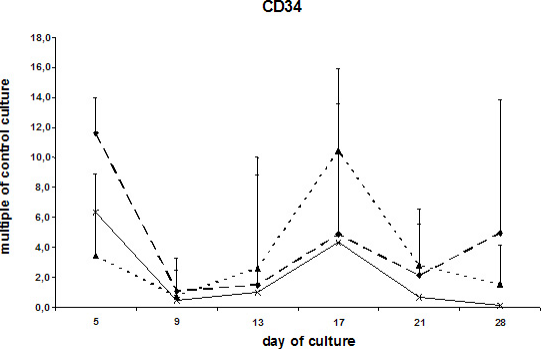
mRNA levels of CD34 expression in ESCs cultured with DAG , BMP-2  and without additional supplements (ESC without LIF) . Data are presented as in Fig. 3.

The transcription of mRNA coding for alkaline phosphatase was slightly increased in cells stimulated with BMP-2. ESCs exposed to DAG did not significantly differ from the control culture (Fig. 5). Expression of osteocalcin, which is regarded as a highly specific marker for osteoblasts, demonstrated showed a double-peaked curve in the DAG-treated cells. ESCs cultured in DAG-supplemented medium showed a prominent peak after 9 days and a second peak beginning at day 17. The first increase was also seen in ESCs cultured in the presence of BMP-2 or the absence of LIF. Interestingly, in all the differentially treated cells a second peak of osteocalcin transcription was observed 7 days later (Fig. 6). All three ESC cultures showed similar expression pattern of the hematopoetic stem cell marker CD34 (Fig. 7).

## Discussion

Currently, there are many efforts to establish cell-based strategies in bone tissue engineering. ESCs are one of many different cell populations, which are being tested for their feasibility for these treatment options. The purpose of this investigation was to determine which supplements in culture medium are most suitable to initiate osteogenic differentiation in ESC cultures. In addition, we investigated the kinetics of gene expression during *in vitro *differentiation.

The results of our microscopical analysis revealed that ESCs cultured in the presence of DAG show by far the highest extent of mineralisation as determined by the occurrence of calcium-phosphate-containing crystals. With respect to extracellular matrix maturation and mineral deposition as crucial steps in the osteogenic cascade [34], DAG seems to be the most promising supplement for inducing osteogenic differentiation in ESCs. In accordance with our microscopical results, a strong increase of collagen I expression was observed at day 11 in the DAG-treated cells. Stimulation with BMP-2 also increased collagen synthesis. Expression of osteocalcin mRNA followed a different pattern and appeared as a double-peaked curve, when ESCs were supplemented with osteogenic agents (DAG or BMP-2). However, the peak induction of osteocalcin mRNA in the BMP-2-treated cells was lower and delayed as compared to DAG-exposed cells. Taken together, these results support the use of DAG as a potent agent for inducing *in vitro *differentiation of ESCs into osteoblast-like cells.

There are only few reports addressing osteogenic differentiation of ESCs published in the literature so far [18,25,34,35]. In agreement with these results we describe here that mineralisation is microscopically evident as early as two weeks of culture. Buttery and co-workers also used DAG as a culture supplement and found that mineralisation was detectable when dexamethasone was added only at day 14 or later [35]. By following this protocol the differentiation process was delayed as compared to the findings in our ESC cultures. While Buttery used only microscopical methods for studying osteogenic differentiation, zur Nieden and colleagues performed also gene expression analyses for osteogenic markers [34]. With respect to the time-course of gene expression with an early increase of collagen I and a later increase of osteocalcin transcripts, their data are comparable to our findings as shown above. Unlike to the findings of zur Nieden and colleagues, an early peak of osteocalcin expression and a minor increase of osteopontin were found in the presented study. The differences could be explained by different concentrations of supplements used for cell differentiation. Zur Nieden et al. used 1,25-OH vitamin D_3 _instead of dexamethasone. According to Zhang et al. vitamin D_3 _increases osteopontin expression in osteoblasts and inhibits expression of osteocalcin [36]. Chaudhry and co-workers replaced dexamethasone with retinoid acid, which was found to be an inductor of mineralization in three-dimensional scaffolds [25]. Notably, alkaline phosphatase was constitutively expressed at high levels in undifferentiated cells [37]. In this experimental setting the mineralisation process was delayed and was detectable only after day 21. Treatment with DAG appeared to be equal or even superior to BMP-2 stimulation regarding the induction of osteogenic differentiation in ESCs. Other authors have used BMP-2 in combination with osteogenic supplements for this purpose [18,38].

An advantage of using ESCs instead of tissue-derived progenitor cells is that ESCs are immortal and could potentially provide an unlimited supply of differentiated osteoblast and osteoprogenitor cells for transplantation. In contrast to embryonic cells, the proliferative, self-renewal and differentiation capacity of cells derived from adult tissues generally decreases with age [39,40]. One major challenge pointing to the use of ESCs lies in overcoming immunological rejection from the transplant recipient. Interestingly, Burt and colleagues performed ESC transplantation in major histocompatibility complex (MHC)-mismatched mice without clinical or histological evidence of graft-versus-host disease (GVHD) [21]. In addition, recent data indicate that ESCs may allow for a low-risk induction of tolerance not requiring any immunosuppression [41].

In conclusion, ESCs differentiate into osteoblast-like cells *in vitro *when stimulated with DAG and showed a time-dependent induction of osteogenic markers. Thus, stimulation with these agents is suitable to generate a promising cell population used for bone tissue engineering.

## Competing interests

The authors declare that they have no competing interests.

## Authors' contributions

JH conceived the study, calculated the statistics and drafted the manuscript, KB carried out the cell culture and the gene expression analysis, HW helped to perform and evaluate the histological investigations, RD, CN, MO, NK and UM participated in its design and coordination and helped to draft the manuscript. All authors read and approved the final manuscript.
